# Forceps Versus Embryulcia Instruments

**Published:** 1856-06

**Authors:** J. R. Freese

**Affiliations:** Bloomington, Ill.


### ARTICLE II.—*Forceps versus Embryulcia Instruments.* By J. R. FREESE, M.D., Bloomington, Ill.

Most of my friends call me a *bold* operator, a bold practitioner, &c.; nevertheless, I know full well, that, however bold I may be where I can see and feel, and thus know exactly what I am doing, I am considerably timid when it comes to working with sharp instruments among delicate organs and tissues in the dark.

There is no operation to which I have ever felt a greater aversion than craniotomy, or embryotomy. Not that I have any conscientious scruples about it, but the delicacy of the occasion; the soft, delicate tissues with which the instruments must come in contact more or less; and the ugly look of the instruments to be used, have all conspired to make me wish that I may never be under the necessity of performing the operation; and thus far I have been gratified in my wish.

A few months ago, however, I thought (to use a common expression) that I "was in for it"—and the manner in which I "got out of it," will form the subject of this article. If the publishing of it enables any of my medical brethren to get out of a like difficulty in a like satisfactory manner, I shall consider myself fully repaid for the trouble of reporting the case.

Mrs. W. was, and is, a strong, healthy woman, of about 32 years of age. She was not married until 30, and in less than a year thereafter she was in the usual interesting condition, and prepared to present her husband with a pledge of her affection. Her labor pains commenced on Friday evening, and continued, with more or less force, until Sunday night at 12 o'clock, when I was sent for as consulting physician. On arriving at the house, and learning the history of the case from the attending physician, I proceeded, at once, to make a careful examination *per vaginum*, and found, somewhat to my horror, that the antero-posterior diameter of the pelvis was only from two to three inches, while the transverse diameter was only from three to four inches. (My estimates of size were made only by the fingers—not having any better means at hand—yet I think them correct, or about so.) At once the horrors of craniotomy, with its punctures, and breakings, and crushings, and mutilations, arose to my mind; but, not having any embryulcia instruments with me, yet having brought along a pair of Hodge's forceps, and feeling that whatever was done would have to be done quickly, I concluded to try the efficacy of a milder kind of crushing, before going into the wholesale business of craniotomy. As the woman's pulse was fast sinking—indeed, her pulse was scarcely perceptible—I at once fixed upon the following plan, and resolved to put it into execution without delay:—

The pelvic strait, I concluded, was large enough to admit the entrance and exit of the *closed* forcep blades, and if I could get them fairly on the head of the child (it being a vertex presentation,) I would use force enough to *crush the head within the size* of the forcep blades, and thus deliver the woman. Having told the attending physician of my plan, and informed the friends that the operation was a critical one for the mother, and certainly fatal to the child, I proceeded to place the woman *in situ*, and apply the blades of the forceps *secundum artem.* Having got the blades fairly applied, and assured myself that there was no portion of the genital organs within their grasp, I seized and pressed the handles of the forceps with such force as to bring them directly together, aud thus secured them with a cord. I was then prepared to draw upon the fœtus, which I did first very gently, with a slight rotary movement, and then with considerable force, but not an iota did it come forward. After allowing my patient to rest for a few moments, I again applied force, and a very considerable too; and this time there was a slight advance, sufficient to encourage me to make a third attempt, after allowing my patient a little time to rest. I now changed her position from the edge to the middle of the bed, and had her hips raised by placing pillows underneath. I stood myself upon the bed, and, when all was ready, I again applied a very considerable force to the forceps, giving them such motion and direction as would favor the delivery; and, as "fortune favors the brave," and "perseverance is followed by success," this time I succeeded in completely delivering the head. I immediately untied the handles and took the forceps off from the head—and such a looking head! two or three times as long as it was broad, looking more like a section of the human arm, than a fœtal head. I speedily pressed and molded it into shape as best I could, and then proceeded to deliver the balance of the body, which I had to do entirely by force, as there were no labor pains whatever. Observing that the child did not breathe, I blew into its mouth and tried to make a bellows of its chest, and pretty soon my efforts were rewarded by seeing the child commence to breathe. Having cut the cord, I passed the child to the nurse, with particular directions as to its care.

The mother did not flow more than usual; and, except very great exhaustion, consequent upon the long labor and operation, no special symptoms presented themselves to our care. In nine days she was up and about the house, attending to her usual duties.

Strange to say, and contrary to every probability, and my own definite prognosis, *the child lived*—continued to enjoy good health; and, to-day, is as healthy, and seems to enjoy as good mental faculties as any child in the city!

Now, for the lessons which we should learn from this case; which, to me have been, and to others, I think, will be, interesting and instructive.

According to most authorities, a man would be justifiable in proceeding, at once, with embryotomy, whenever he found the bones of the pelvis approach nearer each other than three inches in the antero-posterior diameter by four inches transversely; *especially*, where the patient had been in strong labor more than *two whole days*, and the examination indicated the child to be *over the usual size*, as it proved in this instance. Had I proceeded at once with craniotomy, the child, of course, would have been destroyed; and the severity and tediousness of the operation, in her then enfeebled condition, would, nine chances in ten, have produced the death of the mother; both of which unhappy consequences were avoided by the expedient I adopted.

Again, it is another instance in proof of the great amount of *pressure* and *distortion* which the fœtal head will bear without destroying life, or injuring the mental faculties; and it teaches us, that, even in the most desperate cases, we should not despair of the life, either of mother or child. I frankly confess, that when I took off the forceps and pressed the child's head into shape, it was not with any idea of the child's living, or of saving its life, but only to give it a more comely look to the ladies present, that they might not be horrified at the unseemly distortion, which the pressure of the forceps had produced: and when I produced artificial respiration, it was more as a "*professional ruse*" than from any hope of benefit—nevertheless, I did them, and the result proved the fallacy of my own definite prognosis, much, however, to my own satisfaction. Such mistakes in prognosis, don't hurt the doctor much.

Another lesson to be learned from this case, is that of *perseverance.* If I had become discouraged, and given up my plan of treatment, after completely failing in the first attempt; or, if I had given up after the second attempt, both lives would have been lost: for I think now, as I thought then, that the mother's life would have been certainly sacrificed in the operation of craniotomy, however skilfully it might have been performed. I persevered, and, in the third attempt, I succeeded, although I had failed twice previous. One line of the school-boy's song reads:—

"If at first you don't succeed, try, try again."

And it is a lesson to be heeded by the surgeon, as well as by the school-boy.

The *force* that may be applied with safety, in extracting the fœtus, is another lesson to be learned from this case. I am a strong, muscular man—and when I say that I applied a "very considerable force," you may well guess what it means. However, before applying such great force, I remembered to observe Davy Crockett's axiom, to "be sure I was right, before going ahead." I was sure that there was sufficient room for the exit of the closed forcep blades, and that there was no portion of membrane, or other portion of the genital organs, within the grasp of the forceps; and, as I felt that life or death depended on the success of my plan, I applied more force than I would have dared to do under less urgent circumstances. The woman, in recovering, did not complain of more than ordinary soreness, and no immediate or subsequent ailment has happened to the womb or vagina on account of the force applied.

In conclusion, let me suggest, that, in all cases where there is room for the entrance and exit of the closed forcep blades, try my expedient; and if you succeed, as I did, you'll thank me for the suggestion.

I look upon such fortunate results to exceeding difficult cases, as green spots in the great desert of practice; and, if others of our professional brethren will take the trouble to report such as have occurred in their own practice, it will relieve the *tedium* of, and cheer us on, in the arduous duties of our noble profession.

